# Comparison of Students' Mental Wellbeing, Anxiety, Depression, and Quality of Life During COVID-19's Full and Partial (Smart) Lockdowns: A Follow-Up Study at a 5-Month Interval

**DOI:** 10.3389/fpsyt.2022.835585

**Published:** 2022-04-20

**Authors:** Muhammad Aqeel, Tasnim Rehna, Kanwar Hamza Shuja, Jaffar Abbas

**Affiliations:** ^1^Foundation University Islamabad, Rawalpindi Campus, Islamabad, Pakistan; ^2^National University of Modern Languages, Islamabad, Pakistan; ^3^School of Media and Communication, Shanghai Jiao Tong University, Shanghai, China; ^4^Antai College of Economics and Management, Shanghai Jiao Tong University, Shanghai, China

**Keywords:** mental health, anxiety, depression, quality of life, COVID-19 full lockdown, smart lockdown, COVID-19 preventive health behavior

## Abstract

**Objective:**

Scholars have debated the COVID-19's full and partial lockdowns' effectivity to control the transmission of the new case. They emphasized the provision of required economic and social resources worldwide. Past literature related to COVID-19 has contributed little evidence to examine the efficacy of full and partial lockdown measures with experimental perspectives at different intervals. This study bridges this literature gap and explores the full and smart lockdowns' impacts on Pakistani students' mental health, depression, quality of life, and anxiety symptoms, during the various waves of the COVID-19 pandemic.

**Method:**

This pretest and posttest experimental designed web-based survey recruited 40 students from March 23 to August 23, 2020, and recorded their responses. The study incorporated four standardized psychological instruments to receive the desired datasets related to students' mental health, quality of life, anxiety, and depression. Researchers shared data links with the participants via social media, WhatsApp. The study applied one-way and multivariate ANOVA tests (analysis of variance) to draw the desired results.

**Results:**

This study's findings suggest that both full and partial COVID-19 lockdowns effectively improve students' mental health and quality of life. These measures help reduce anxiety and depressive symptoms among university students. The study results exhibit that partial lockdown (PL) is more effective in improving quality of life. Besides, PL helps reduce anxiety symptoms than complete lockdown among Pakistani students.

**Conclusion:**

The present study's findings suggest that students are vulnerable. They need particular interventions and preventive measures to protect and improve their mental health and quality of life during a global pandemic. As the stressful experience of the epidemic persists in Pakistan. It will also be interesting to examine the psychological impact of the successive waves of the COVID-19 pandemic.

## Introduction

The epidemic of coronavirus infections (COVID-19) has been extensively affecting the living and life of individuals globally, more specifically after the statement of an international epidemic through the World Health Organization (WHO) in the month of March 2020 (1–5). There were approximately 6.91 million people infected with the COVID-19 in June 7, 2020 across the world (6, 7). Therefore, several countries of the world, such as United Kingdom, United States of America, France, Russia, India, and Pakistan, executed a variate of anti-epidemic tools, including the shutdown of private and public places, closing down the complete transit system, and limiting travel for overseas nationals to prevent the transmission of the extremely transmissible virus from people-to-people (6–14).

Pakistan confirmed its first coronavirus disease (COVID-19) case on February 26, 2020. Pakistan, like several other nations, implemented the full lockdown plan into effect on March 23, 2020, to ensure “social distance” by “home quarantine” to prevent the transmission of the extremely contagious virus in its populace (15–20). Conversely, all publican and private schools were shut down first from March 23 to April 15, 2020 all over the country. However, given the complex economic situation of the country, the government converted the complete lockdown into “partial lockdown” on May 9, 2020 (15). After rigorous evaluation of the critical situation of COVID-19 in Pakistan, partial lockdown was extended to May 31, 2020, and later prolonged to August 15, 2020 in the first wave of the COVID-19 epidemic (18, 21–24), but it was finally ended on September 15, 2020 (21).

This exceptional experience of home-based quarantine during full and partial lockdown with the uncertainty of professional and academic career has complicated effects on the psychological wellbeing of university students (3, 4). For instance, a similar research conducted to explore the influence of home-based quarantine later on the serious acute respiratory syndrome epidemic established a relationship between prolonged time of home-based quarantine with a greater level of depression and anxiety in students (1–4, 18, 25).

The current epidemic of COVID-19 is developing a psychosocial chaotic condition in Pakistan like many other countries that have been experiencing a rapid increase in psychological disorders such as fear, sleep, stress, depression, anxiety disorder, substance use, and suicidal behavior in people (26–37). The results of many previous studies conducted in China revealed that the higher the disclosure of “misinformation” by social media, the more probability it is in contributing to the growth of depression, anxiety, and other psychological problems in students (14, 38, 39). Many similar studies also revealed that there is a significant reduction in daily social interactions of university students, and they experienced lack of social support due to lockdown. These, along with the occurring stressors related with the current pandemic, can all potentially lead to affect the mental health of students negatively. Earlier studies indicated that similar situations have multiple psychological consequences on the lives of students such as chronic and acute stress, depression (33, 40–44), and reduced quality of life (45–50). A similar study conducted in Chinese students indicated that having an infected relative or acquaintance to COVID-19 is also a potential risk factor for anxiety (51). Whereas, factors, such as stable family income, living in urban area, and living with family, serve as protective factors (51).

In another survey conducted on 8,079 Chinese students with age ranging from 12 to 18 by Zhou et al. (52) stated that there was a significant prevalence of anxiety (37%), depression (43%), and combined symptoms of anxiety and depression (31%) in university students during the first wave of the COVID-19 epidemic (52, 53). A study of similar nature, also recently conducted in Pakistan by Salman et al. (54), revealed that there was positive association among COVID-19 epidemic, anxiety, and depression in university students. Furthermore, the results of this study indicated that university students were found to have moderate to severe anxiety (34%) and depression (45%) during the COVID-19 lockdown (54). Additionally, similar studies demonstrated that there was a negative impact of COVID-19 on mental health, and it also led toward anxiety and depression (9, 55–57). Other pieces of evidence also illustrated that female university students who had poor sleep quality, showed more mental health problems during the COVID-19 lockdown (58–60).

Given the bewildering situations, it is very important to examine and comprehend the psychological experience of students in Pakistan, more specifically in the COVID-19 epidemic. This kind of study is expected to explore the mental health effects of an unpredicted emergency on university students, and to create and implement effective preventions and interventions to mitigate the psychological problems of people. The present research was intended to address and comprehend the mental health problems in Pakistani university students. This study aimed to examine and compare the effect of the full and partial (smart) COVID-19 lockdown experience on mental health, quality of life, symptoms of anxiety, and depression in Pakistani university students during the first wave of the COVID-19 epidemic.

University students have also been extensively affected during the first wave of the COVID-19 epidemic. Most of the universities of the world have been shut down, and university pupils have had to experience drastic changes in their academic and social life (7). More specifically, Italian students have been the first to experience the full lockdown with the closing of educational institutes and the shift to distance education, whereas many other countries' university students were possibly already updated regarding the full and partial lockdown experience. In Pakistan, all educational institutes were first shut down on March 23, 2020, and educational activities including administrative management, degrees, lessons, and exams, have been modified to online sources. The aforementioned activities were taking face-to-face classes through online modalities, using different learning sources, and sharing class notes, such as slides and learning materials (1–4, 61), thus, university students have had to adapt and modify their learning techniques to distance classes (62–65). Furthermore, many of the university students who joined the online classes from outside their homes were forced to go back to their houses abruptly and to spend the full lockdown in their university towns.

So far, there is a few studies conducted that have explored how severe the impact of COVID-19 related lockdown is on the overall mental health and quality of life of the student population (66). The few studies that have inspected the psychological effects of COVID-19 is on the infected population (59, 60, 67, 68). Similarly, a few studies have tried to assess the impact of lockdown itself on student population and changes from pre- to post-outbreak (56). However, no study has seen the effect in Pakistani population where the students experienced two different forms of lockdowns, i.e., a full lock down and a smart lockdown (69). The present study, by considering all the factors, have tried to investigate the effect of COVID-19 lockdown on psychological, mental health, quality of life, anxiety, and depression in Pakistani students in different phases of lockdown, including complete lockdown and partial lockdown. Additionally, the study also endeavors in assessing mental health, quality of life, anxiety, and depression in those students who have preexisting mental health issues.

## Methodology

### Research Objective and Hypotheses

Based on the aforementioned concerns, this study aimed to explore psychological experiences of university students during both the full and partial (smart) COVID-19 lockdowns in the first wave of the COVID-19 epidemic in Pakistan. Moreover, the effect of the full and partial (smart) COVID-19 lockdown experiences on mental health, quality of life, symptoms of anxiety, and depression in Pakistani university students during the first wave of the COVID-19 epidemic was also examined and compared. More specifically, our study also planned to investigate the following hypotheses that were more closely related to psychological experiences of Pakistani university students: Hypothesis 1 (H1): A higher level of anxiety and depression will be significantly higher during the full lockdown in comparison with partial lockdown in the first wave of the COVID-19 epidemic in Pakistan. Hypothesis 2 (H2): There will be significant improvement in general mental health and quality of life during partial lockdown in comparison with full lockdown in the first wave of the COVID-19 epidemic in Pakistan. Hypothesis 3 (H3): To compare the effect of lockdown on students who have preexisting different mild, moderate, severe level of anxiety and depression on the quality of life and mental health during the full and partial phases of lockdown in the first wave of the COVID-19 epidemic in Pakistan.

### Sample

Forty university students with age range from 18 to 25 (*M* = 21.57, SD = 1.05) years were included in the online survey at the Department of Psychology, Foundation University of Islamabad, Pakistan. This online survey was performed at the last week of March during the first wave of the COVID-19 epidemic lockdown, from March 23 to August 23, 2020. The random sampling technique and pretest–posttest experimental design was applied to collect data from BS 8 class students. A 5-month within-group, pre–post-follow-up experimental design was used to examine and compare the effect of the full and partial (smart) COVID-19 lockdown experience on mental health, quality of life, symptoms of anxiety, and depression in Pakistani university students during the first wave of the COVID-19 epidemic. A web-based survey was used to obtain information related to mental health, quality of life, anxiety, and depression using the Google Form. The link of the data was shared with BS 8 students through social media WhatsApp. Ethical approval from higher authority of Foundation University Islamabad was obtained to perform the study. Written informed consent was also obtained from all participants before starting this study.

These inclusion criteria were applied: (1) those students who were diagnosed with COVID-19, and they were guarantees at their homes during the first wave and (2) those students who attended their regular classes from different cities of Pakistan and had proper internet access to fill the forms. The following exclusion criteria were used: those students who were not diagnosed with COVID-19 and did not have access to fill the forms during the first wave were excluded from this survey. All 40 participants were requested to complete standardized psychological questionnaires in this pre–post-follow-up experimental design web-based survey.

All participants filled up an online survey questionnaire in two different phases, such as the pretesting phase (T-0), which occurred at the time when universities were suddenly closed due to epidemic, through online social media application (WhatsApp). The survey questionnaire targeted many psychological domains including anxiety, depression, general mental health, and quality of life.

After the pretesting phase (T-0), the same 40 participants were asked to fill up the same survey questionnaire for the posttesting phase (T-1), which occurred after 22 days (3 weeks) of full lock down, as the full lockdown was lifted and converted into partial (smart) lockdown after that. Last, after the pretesting phase (T-0) and posttesting phase (T-1), the same set of participants were again asked to fill up the forms (T-2) after 5 months as a follow-up, when the smart lockdown was about to end. All participants filled out an online questionnaire in two phases, including the pretest phase (T-0). The government-imposed T-0 when authorities closed universities suddenly due to the ongoing pandemic and began a complete lockdown. After the pretest phase (T-0), the same 40 participants were asked to complete the same questionnaire for the phase of posttest (T-1) after a complete lockdown of 22 days (3 weeks). After the complete lockdown was lifted, the authorities converted the full lockdown into a partial (smart) lockdown. After the pretest phase (T-0) and the posttest phase (T-1), the same 40 participants were asked to fill out the form (T-2) again 5 months later as a follow-up when the smart lockdown was about to end.

### Lockdown Phase's Detail

**Figure 1:**
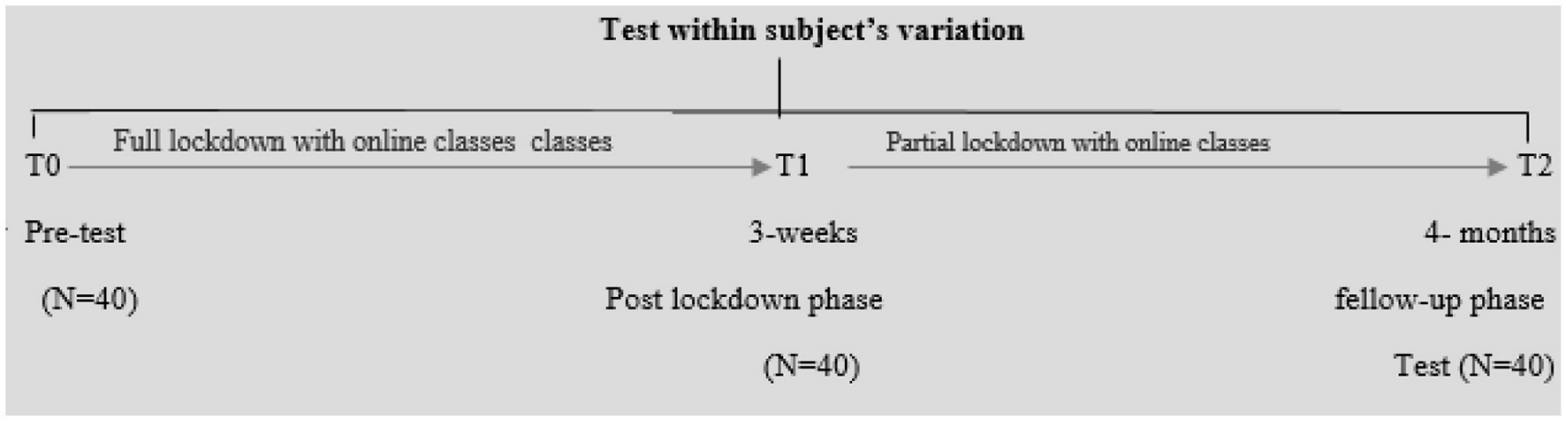
Within group, pretest–posttest design, quasi-experimental research for both full and partial lockdown situations.

The COVID-19 pandemic lockdown occurred in Pakistan in two distinct phases, a complete lockdown, and a partial lockdown. The phases established in the study are based on these lockdown transitions. The first phase, T-0, came into effect on March 23, 2020, when authorities imposed a full lockdown across the country. Phase T-1 took place on April 15, 2020, when the Pakistani government eased lockdown conditions due to severe economic losses. However, given the country's complex financial situation and the rapid increase in infections, the government transitioned from a complete lockdown to a “smart lockdown” from May 9, 2020, to August 15, 2020. It was the time when the study initiated to collect data for phase T-3, as shown in Figure 1.

### Measures

Four standardized instruments were used to measure general mental health, quality of life, depression disorder, and anxiety disorders at the different three phases during the full and partial (smart) lockdown in Pakistan students.

### The Beck Depression Inventory

This instrument (Beck Depression Scale) contains 21 items, and this self-reporting tool helps measure depression severity among psychiatric individuals and healthy populations (70). The B.D.I. item scores are measured on the Likert scale of four points, ranging from 0 to 3 (0 = symptom absent and 3 = severe symptoms). The scores on the BDI-II tool are classified as follows: the B.D.I. scores within the range of 0–13 indicate normal depression, the scores between 14 and 19 show mild depression, and 20–28 scores reflect moderate depression. Similarly, the scores 29–63 show severe depression among people. Similarly, the I.P.Q. R has exemplified passable validity and reliability. The present study shows the Cronbach alphas (α) 0.91, which specifies adequate reliability.

### The Beck Anxiety Inventory

The Beck Anxiety Inventory (BAI) is a self-reporting instrument that comprises the 21 items. This tool helps evaluate the anxiety severity among psychiatric people as well as a healthy population (71). The BAI instrument's each item's score is measured on the Likert scale based on four points ranging from 0 to 3 (0 = not at all and 3 = severely—it bothered me a lot). The classification of the BAI instrument is described as given: the scores 0–7 indicate low or minimal anxiety, the scores 8–15 show mild anxiety level, and the scores 16–25 indicate moderate anxiety, whereas the scores 26–63 show severe anxiety among people. The BAI measurement displayed satisfactory validity and reliability. This study has shown the Cronbach alphas (α) 0.93 that stipulates acceptable reliability.

### The Warwick–Edinburgh Mental Well-Being Scale

The Warwick–Edinburgh Mental Well-being scale (WEMWBS) is a self-reporting tool based on 14 items that help assess the mental wellbeing of ordinary individuals and the clinical population (72). The five-point Likert scale measures WEMWBS item scores that show the following points: 1 = none of the time and 5 = all of the time. The WEMWBS instrument indicated adequate validity and reliability. This current study has shown the Cronbach alphas (α) 0.91 that stipulates acceptable reliability.

### World Health Organization Quality of Life Scale

The World Health Organization Quality of Life Scale (WHOQOL)-BREF is a self-reporting instrument that contains 26 items to measure individuals' quality of life. This tool helps evaluate normal individuals' and the clinical population's quality of life (73). The five-point Likert scale helps measure instrument items based on four subscales. It measures social relationships, environment, and psychological and physical health. The WHOQOL-BREF displayed acceptable validity/reliability. This study exhibits an adequate value of the Cronbach alpha (α = 0.94), which indicates satisfactory reliability.

### Data Management and Analysis Plan

Data were collected using a web-based online survey. After completing the data, the missing values were checked using an imputation technique using SPSS-23 for all the scales used in the study. Before carrying out the analyses, values of all scales were primarily transformed into standardized Z-scores by gathering the pretest, posttest, and follow-up values from all students to compute the mean and standard deviation. One-way analysis of variance (ANOVA) and multivariate analysis were applied to analyze the data of the present study. Baseline subject comparisons have been carried out applying nonparametric statistics such as chi-square test. Moreover, multivariate analysis and chi square analysis have been performed to examine and compare the effect of the full and partial (smart) COVID-19 lockdown experience on mental health, quality of life, symptoms of anxiety, and depression in Pakistani university students during the first wave of the COVID-19 epidemic. The sample adequacy was determined by the value of eta squared **(**η^2^) in the present study. The value of eta squared revealed that sample size was sufficient to perform the present study (74, 75).

### Procedure

This current investigation was carried out in accordance with the ethical standards of The Committee on Publication Ethics (COPE). This study was also approved by the Department of Psychology, Foundation University Islamabad, Pakistan. Forty university students living in Pakistan contributed to this pre–post-follow-up experimental design web-based survey. Four standardized psychological instruments were used to obtain information related to mental health, quality of life, anxiety, and depression using Google Forms between March 23 and August 23, 2020. Ethical approval from the higher authority of Foundation University Islamabad was obtained to perform the study. Written informed consent was also obtained from all participants before starting this study. One-way analysis of variance (ANOVA) and multivariate analysis were applied to analyze the data of the present study.

### Consort

**Figure 2:**
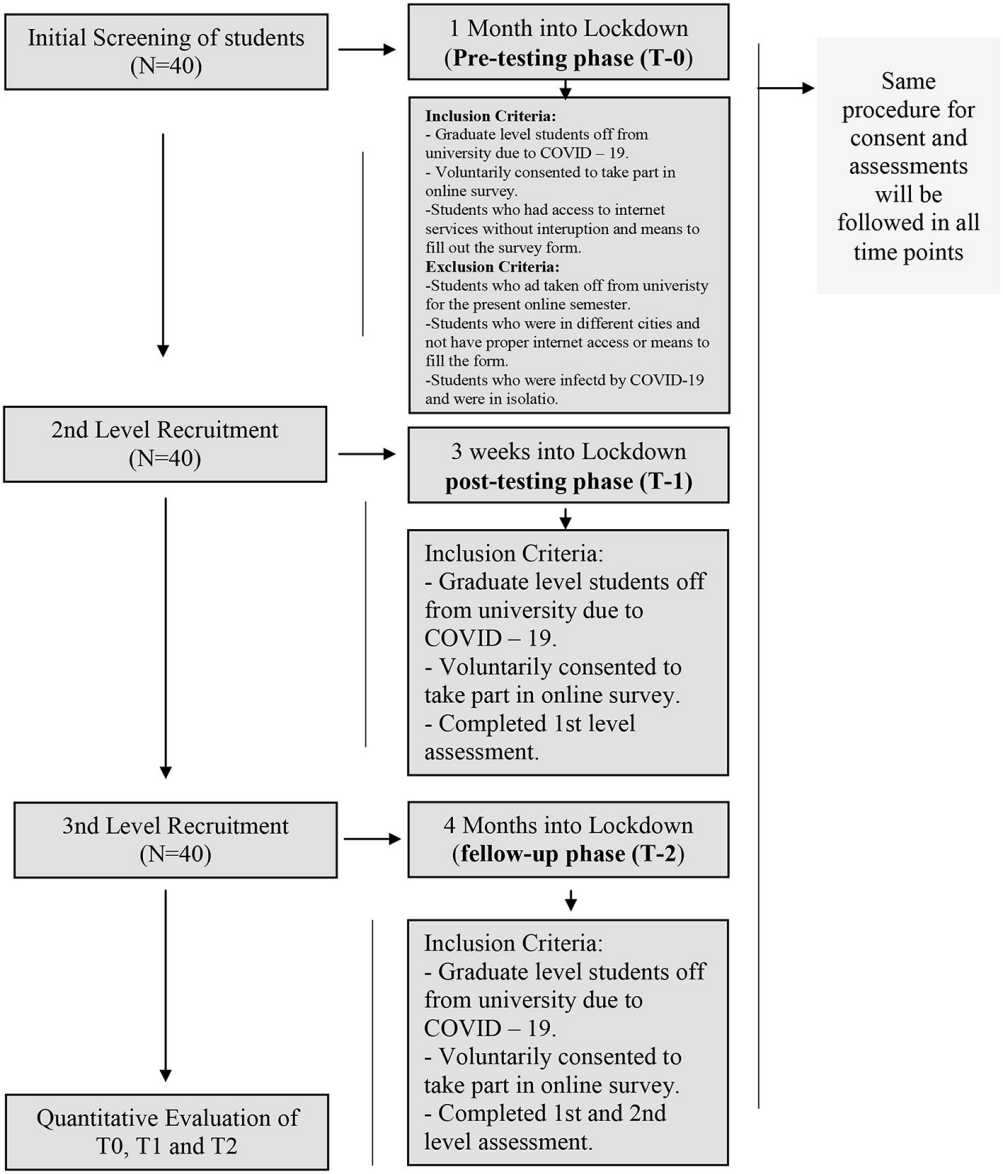
Study flow chart of activities. Illustrative within-group, pretest–posttest design, quasi-experimental research for both full and partial lockdown situations.

The Consolidated Standards of Reporting Trial has been applied for reporting a pretest–posttest design, quasi-experimental research for both full and partial lockdown situations. In the current experimental study, 40 students took part in the study. All participants were enrolled at the Department of Psychology, Foundation University of Islamabad, Pakistan. The flowchart of students with demographic information is presented in Figure 2.

## Results

In **Table 2**, findings of repeated measures ANOVA demonstrated that significant differences were found only on quality of life (*F* = 426.98, *p* > 0.000) between the pretesting phase (T-0), posttesting phase (T-1), and follow-up phase (T-2) in Pakistan students during the COVID-19 epidemic lockdown. On the contrary, no significant differences were found on mental health and development of psychological issues, such as anxiety and depression disorder between pretesting phase (T-0), posttesting phase (T-1), and follow-up phase (T-2) in Pakistan students during the COVID-19 epidemic lockdown. The time period between the pretesting phase (T-0) and posttesting phase (T-1) was assumed as full COVID-19 epidemic lockdown for students. On other hand, the same data from the posttesting phase (T-1) and follow-up phase (T-2) were considered as partial COVID-19 epidemic lockdown for Pakistan students. Findings of the study revealed that participants exposed significant improvement in the quality of life in the three phases. Moreover, eta squared **(**η^2^) was used to examine the adequacy of effect size for the present study sample. The value of eta squared **(**η^2^ = 0.99) shows medium effect size in the present study. The value of eta squared revealed that the sample was sufficient to perform this study (74, 75).

This study's findings demonstrated that in the first phase of lockdown in Pakistan (*T* = 0), when all the universities were closed, students reported significant decline in quality of life at the starting period of the COVID-19 epidemic, while in phase 2 (*T* = 1), when full lockdown was opened and became more lenient after 3 weeks, participants demonstrated more improvement in quality of life. On the other hand, in phase 3 (*T* = 2), when the university had their semester exams during partial lockdown, participants illustrated more slight improvement in the quality of life. The study findings indicated that full lockdown may be considered more appropriate to improve quality of life, depression disorder, and mental health compared with partial lockdown during the COVID-19 epidemic. Furthermore, findings of the study explained that full lockdown had shockingly enhanced anxiety disorders in university students, whereas in partial lockdown, although there was a slight significant improvement in quality of life, shockingly, mental health decreased, and anxiety and depression disorders both increased during the epidemic crisis.

The aim of the present study was to evaluate the impact of the COVID-19 epidemic lockdown on the interaction between level of anxiety and quality of life of Pakistan students. This study was carried out to examine the effectiveness of the COVID-19 epidemic lockdown for university students through three different phases of COVID-19 epidemic lockdown (pre, post, follow-up) in Pakistan context.

Within-subjects repeated measures ANOVA were carried out to examine the effect of the COVID-19 epidemic lockdown on anxiety, depression, quality of life, and mental health in university students based on three different phases including pre, post, and follow up (A follow-up at 5 months during the COVID-19 epidemic lockdown), in which the three phases were considered as independent variable (IV), while anxiety, quality of life, and mental health were considered as dependent variables (DVs). Results of two-way repeated measures ANOVA revealed significant differences in the three phases on quality of life, mental health, anxiety, and depression in Pakistan students during the COVID-19 epidemic lockdown. Moreover, eta squared **(**η^2^) was used to examine the adequacy of effect size for the present study sample. The value of eta squared **(**η^2^ = 0.99) shows medium effect size in the present study. The value of eta squared revealed that the sample was sufficient to perform this study (74, 75).

### The Effect of the COVID-19 Epidemic Lockdown on Interaction Between Anxiety Level and Quality of Life

**Figure 3:**
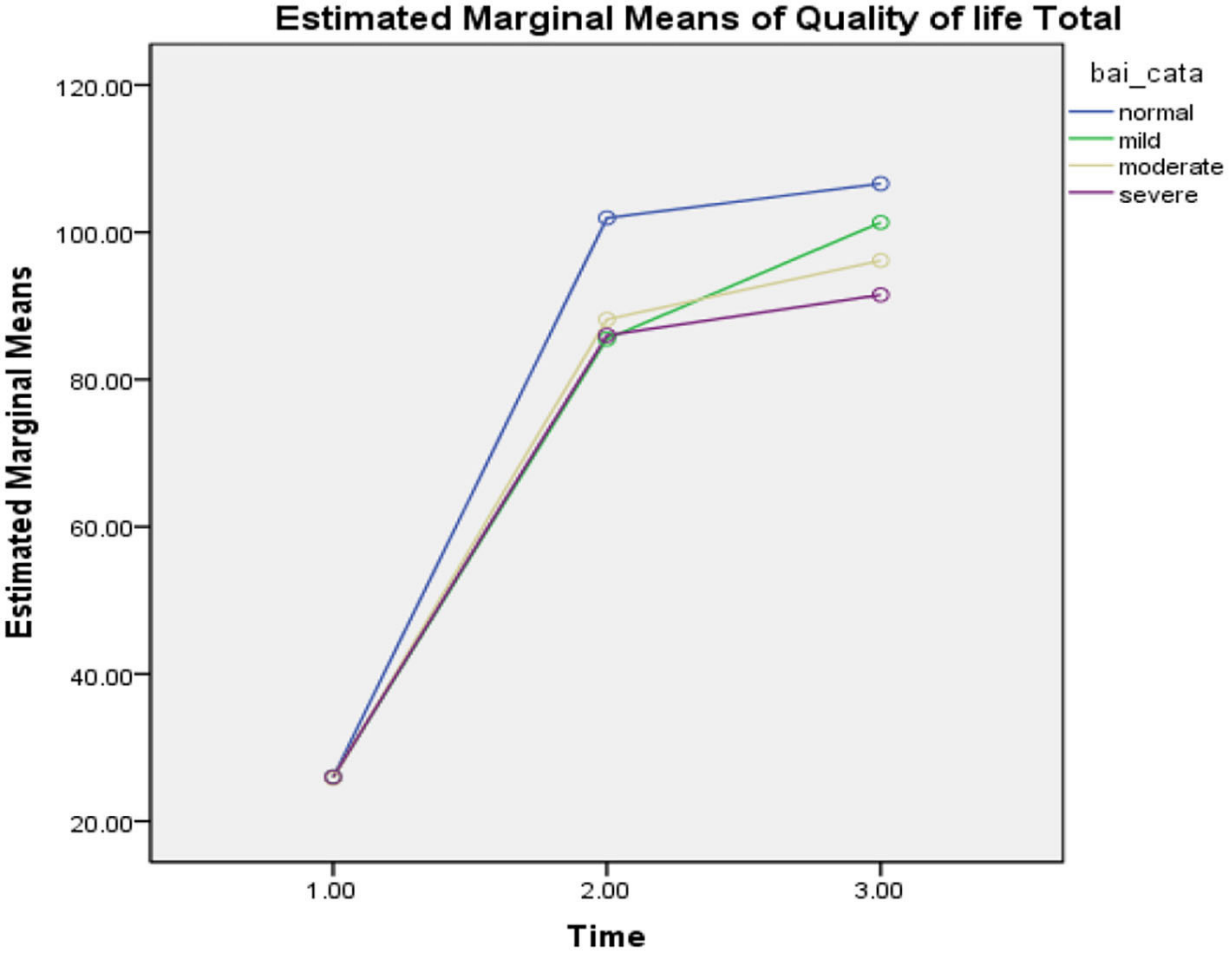
Mean difference of quality of life between T-0 (pretesting phase), T-1 (posttesting phase), and T-2 (follow-up phase) with different levels of anxiety in Pakistan students during the COVID-19 epidemic lockdown (*N* = 40). Time, three phases; bai_cata, severity level of anxiety; quality of life total, quality of life.

In **Table 3**, repeated measures ANOVA was used and displayed significant effect of the COVID-19 epidemic lockdown on the quality of life in the three phases (*F* = 215.35, *p* = 0.000), level of anxiety (BAI) (*F* = 2.99, *p* = 0.12), and interaction effect between the level of anxiety and the three phases (*F* = 1.94, *p* = 0.08, $\eta_{p}^{2}$ = 1.99). The findings of the study revealed that participants exposed significant improvement in the quality of life at the three phases (see Figure 3).

In the first phase of the lockdown in Pakistan (*T* = 0), when all universities were closed due to the COVID-19 epidemic, participants reported significant decline in quality of life with different levels of anxiety as normal (*M* = 25.94, SD = 0.23, *n* = 18), mild (*M* = 26.00, SD = 0.02, *n* = 8), moderate (*M* = 25.80, SD = 0.44, *n* = 5), and severe (*M* = 26.00, SD = 0.01, *n* = 9), while in phase 2 (*T* = 1), participants demonstrated more improvement in the quality of life with different levels of anxiety, such as normal (*M* = 101.93, SD = 13.01, *n* = 16), mild (*M* = 85.50, SD = 24.14, *n* = 8), moderate (*M* = 88.16, SD = 9.70, *n* = 6), and severe (*M* = 86.00, SD = 13.78, *n* = 10). On the other hand, in phase 3 (*T* = 2), when the university was conducting their semester exams, participants illustrated more slight improvement in the quality of life with higher level of anxiety, such as normal (*M* = 106.60, SD = 10.79, *n* = 15), mild (*M* = 101.33, SD = 15.82, *n* = 9), moderate (*M* = 96.16, SD = 8.70, *n* = 6), and severe (*M* = 91.50, SD = 13.35, *n* = 10). In the table above, the findings of the study revealed that those participants who had reported moderate and severe levels of anxiety, had lower level of quality of life compared with those who had normal and mild level of anxiety, who were also found to have a higher level of quality of life during the COVID-19 epidemic lockdown in Pakistan (see Figure 3).

### The Effect of the COVID-19 Epidemic Lockdown on Interaction Between Depression Level and Quality of Life

**Figure 4:**
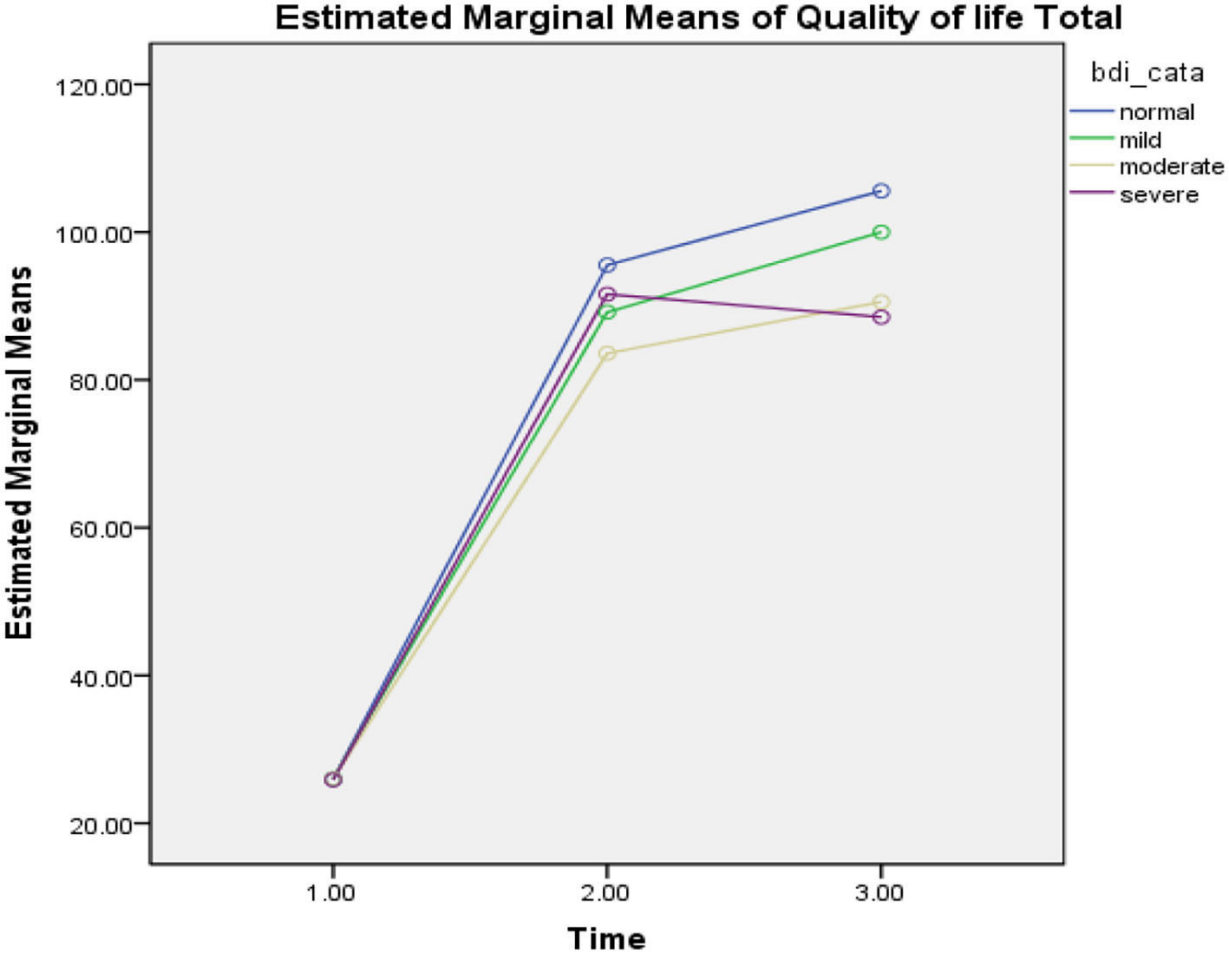
Mean difference of quality of life between T-0 (pretesting phase), T-1 (posttesting phase), and T-2 (follow-up phase) with different levels of depression in Pakistan students during the COVID-19 epidemic lockdown (*N* = 40). Time, three phases; bdi_cata, severity level of depression; quality of life total, quality of life.

Repeated measures ANOVA was carried out and illustrated the significant effect of the COVID-19 epidemic lockdown on the quality of life in the three phases (*F* = 210.46, *p* = 0.000), level of depression (BDI) (*F* = 2.82, *p* = 0.12), and interaction effect between level of depression and the three phases (*F* = 1.21, *p* = 0.30, $\eta_{\text{p}}^{2}$ =0.98). The findings of the study revealed that participants had a significant improvement in the quality of life at the three phases (see Figure 4).

In the first phase of the lockdown in Pakistan (*T* = 0), when all universities were closed due to the COVID-19 epidemic, participants reported a significant decline in the quality of life with different levels of depression such as normal (*M* = 25.96, SD = 0.20, *n* = 25), mild (*M* = 26.00, SD = 0.00, *n* = 4), moderate (*M* = 26.00, SD = 0.00, *n* = 4), and severe (*M* = 25.85, SD = 0.37, *n* = 7), while in phase 2 (*T* = 1), participants demonstrated more improvement in the quality of life with different levels of depression such as normal (*M* = 95.54, SD = 19.28, *n* = 24), mild (*M* = 89.16, SD = 10.98, *n* = 6), moderate (*M* = 83.60, SD = 5.94, *n* = 5), and severe (*M* = 91.60, SD = 17.05, *n* = 5). On the other hand, in phase 3 (*T* = 2), when the university was conducting their semester exams, participants illustrated more slight improvement in quality of life with higher level of depression such as normal (*M* = 105.58, SD = 11.38, *n* = 24), mild (*M* = 100.00, SD = 7.00, *n* = 3), moderate (*M* = 90.55, SD = 13.29, *n* = 9), and severe (*M* = 88.50, SD = 13.22, *n* = 4). In the table above, the findings of the study revealed that those participants who had moderate and severe levels of depression, were reported to have a lower level of quality of life compared with those who had normal and mild level of depression, who were also found to have a higher level of quality of life during the COVID-19 epidemic lockdown in Pakistan (see Figure 4).

### The Effect of the COVID-19 Epidemic Lockdown on Interaction Between Anxiety Level and Mental Health

**Figure 5:**
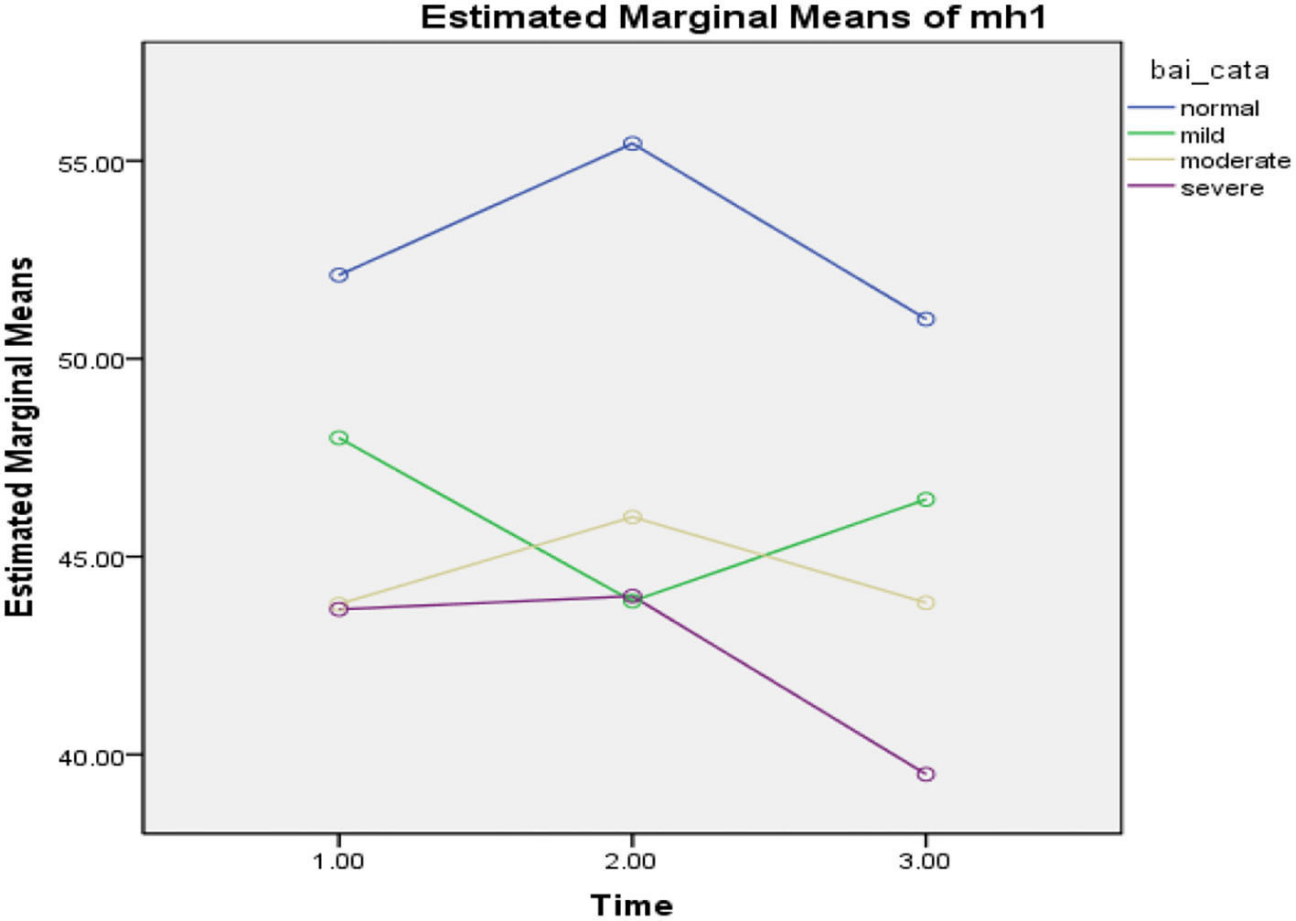
Mean difference of mental health between T-0 (pretesting phase), T-1 (posttesting phase), and T-2 (follow-up phase) with different levels of anxiety in Pakistan students during the COVID-19 epidemic lockdown (*N* = 40). Time, three phases; bai_cata, severity level of anxiety; quality of life total, quality of life.

Repeated measures ANOVA was applied and showed a significant effect of the COVID-19 epidemic lockdown on mental health in the three phases (*F* = 1.02, *p* = 0.39), level of anxiety (BAI) (*F* = 19.81, *p* = 0.002), and interaction effect between level of anxiety and the three phases (*F* = 0.40, *p* = 0.87, $\eta_{\text{p}}^{2}$ = 0.99). The findings of the study demonstrated that participants had a significant decline in mental health having different levels of anxiety at the three phases (see Figure 5).

In the first phase of the lockdown in Pakistan (*T* = 0), when all universities were closed due to the COVID-19 epidemic, the participants reported a significant decline in mental health with different levels of anxiety as normal (*M* = 52.11, SD 11.22, *n* = 18), mild (*M* = 48.00, SD = 5.83, *n* = 8), moderate (*M* = 43.80, SD 8.01, *n* = 5), and severe (*M* = 43.66, SD = 10.94, *n* = 9), while in phase 2 (*T* = 1), participants demonstrated more decline in mental health with the different levels of anxiety, such as normal (*M* = 55.43, SD = 8.86, *n* = 16), mild (*M* = 43.87, SD = 13.62, *n* = 8), moderate (*M* = 46.00, SD = 7.23, *n* = 6), and severe (*M* = 44.00, SD = 5.49, *n* = 10). On the other hand, in phase 3 (*T* = 2), when the university conducted their semester exams, participants illustrated more decline in mental health with higher level of anxiety such as normal (*M* = 51.00, SD = 10.74, *n* = 15), mild (*M* = 46.44, SD = 13.63, *n* = 9), moderate (*M* = 43.83, SD = 5.56, *n* = 6), and severe (*M* = 39.50, SD = 7.41, *n* = 10). In the table above, the findings of the study revealed that those participants who had reported moderate and severe level of anxiety were found to have a lower level of mental health compared with those who had normal and mild levels of anxiety, who were also found to have higher level of mental health during the COVID-19 epidemic lockdown in Pakistan (see Figure 5).

### The Effect of the COVID-19 Epidemic Lockdown on Interaction Between Depression Level and Mental Health

**Figure 6:**
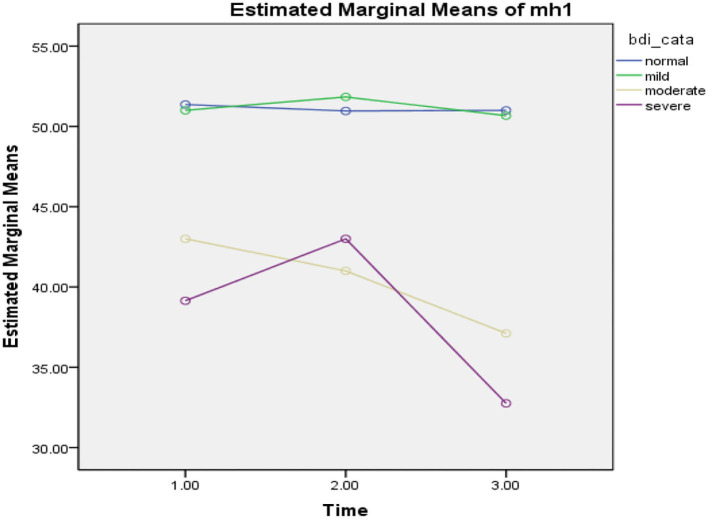
Mean difference of mental health between T-0 (pretesting phase), T-1 (posttesting phase), and T-2 (follow-up phase) with different levels of depression in Pakistan students during COVID-19 epidemic lockdown (*N* = 40). Time, three phases; bdi_cata, severity level of depression; quality of life total, quality of life.

Repeated measures ANOVA was applied and showed a significant effect of the COVID-19 epidemic lockdown on mental health in the three phases (*F* = 1.90, *p* = 0.18), level of depression (BDI) (*F* = 24.31, *p* = 0.000), and interaction effect between level of anxiety and the three phases (*F* = 0.49, *p* = 0.80, $\eta_{\text{p}}^{2}$ = 0.99). The findings of the study demonstrated that participants showed a significant decline in mental health having different levels of depression at the three phases (see Figure 6).

In the first phase of the lockdown in Pakistan (*T* = 0)m when all universities were closed due to the COVID-19 epidemic, the participants reported a significant decline in mental health with different levels of depression as normal (*M* = 51.36, SD = 10.44, *n* = 25), mild (*M* = 51.00, SD = 6.37, *n* = 4), moderate (*M* = 43.00, SD 7.25, *n* = 4), and severe (*M* = 39.14, SD = 6.96, *n* = 7), while in phase 2 (*T* = 1), participants demonstrated more decline in mental health with different levels of depression such as normal (M 50.95, SD = 11.81, *n* = 24), mild (*M* = 51.83, SD = 4.30, *n* = 6), moderate (*M* = 41.00, SD = 4.52, *n* = 5), and severe (*M* = 43.00, SD = 6.36, *n* = 5). On the other hand, in phase 3 (*T* = 2), when the university conducted their semester exams, participants illustrated more decline in mental health with a higher level of depression such as normal (*M* = 51.00, SD = 9.13, *n* = 24), mild (*M* = 50.66, SD = 2.30, *n* = 3), moderate (*M* = 37.11, SD = 6.19, *n* = 9), and severe (*M* = 32.75, SD = 9.42, *n* = 4). In the table above, the findings of the study revealed that those participants who had reported moderate and severe level of depression, were found to have a lower level of mental health compared with those who were found with normal and mild level of depression, who were also found to have a higher level of mental health during the COVID-19 epidemic lockdown in Pakistan (see Figure 6).

## Discussion

The present research aimed to explore psychological experiences of university students during both the full and partial (smart) COVID-19 lockdowns in the first wave of the COVID-19 epidemic in Pakistan. More particularly, the effect of the full and partial (smart) COVID-19 lockdown experiences on mental health, quality of life, symptoms of anxiety, and depression in Pakistani university students during the first wave of the COVID-19 epidemic was also examined and compared. Additionally, the effect of lockdown on students who had preexisting different mild, moderate, severe levels of anxiety, and depression on the quality of life and mental health during the full and partial phases of lockdown in the first wave of the COVID-19 epidemic in Pakistan was compared. This study's findings revealed that both the full and partial COVID-19 epidemic lockdown were considered effective in improving mental health and quality of life or reducing symptoms of anxiety and depression in Pakistani university students. Furthermore, this study revealed that partial lockdown is more effective in improving the quality of life and reducing symptoms of anxiety in comparison with the full lockdown in a sample of Pakistani students. The results of the present study supported our study objectives and hypotheses. The results of the present study supported the findings of previous studies (26–39, 76). The current epidemic of COVID-19 is developing a psychosocial chaotic condition in Pakistan like many other countries that have been experiencing a rapid increase in psychological disorders such as fear, sleep, stress, depression, anxiety disorder, substance use, and suicidal behavior in people (26–37). The COVID-19 epidemic has been extensively affecting the life of university students, and it has not only brought severe medical related issues but also has caused a lot of mental health issues mostly due to lockdown (6–14, 17, 18, 77). Moreover, previous studies illustrated that the COVID-19 epidemic has a detrimental effect on the psychological wellbeing of people globally (11, 18, 78, 79). Most of the earlier studies revealed that the university student's population was considered one of the most vulnerable population during the first wave of the COVID-19 epidemic. University students have severe mental health problems as a result of the partial and full lockdown due to closure of educational institutes. It is considered that peer influence and interaction with teachers play an important positive role in reducing their mental health problems including stress, anxiety, and depression. It also helps them to improve mental health and quality of life in university students. Furthermore, it helps them cope with their personal issues in student life, their social networking, and interactions with teachers (16, 80). However, due to COVID-19 outbreak, many countries in the world shut down all their educational institutes. Like other countries, Pakistan was also facing a similar issue and shut down all academic institutes to prevent transmission of the contagious virus (18, 54). However, unlike other countries, the nature of the lockdown in itself was unique in Pakistan as majority of the countries opted for a full lockdown. In Pakistan, due to the economic condition, a partial lockdown was employed in the latter half (21). University students of Pakistan also faced and reported deleterious mental health and health issues (16, 18). Many similar studies also revealed that there is a significant reduction in the daily social interactions of university students, and they experienced lack of social support due to lockdown. These, along with the occurring stressors related with the current pandemic, can all potentially lead to affect the mental health of students negatively. Earlier studies indicated that similar situations have multiple psychological consequences on the lives of students such as chronic and acute stress, depression (33, 34, 37, 39–44, 53), and reduced quality of life (45–47). A similar study conducted in Chinese students indicated that having an infected relative or acquaintance can also be a potential risk factor for anxiety (51), whereas factors like stable family income, living in an urban area, and living with family served as protective factors (51).

**Figure 7:**
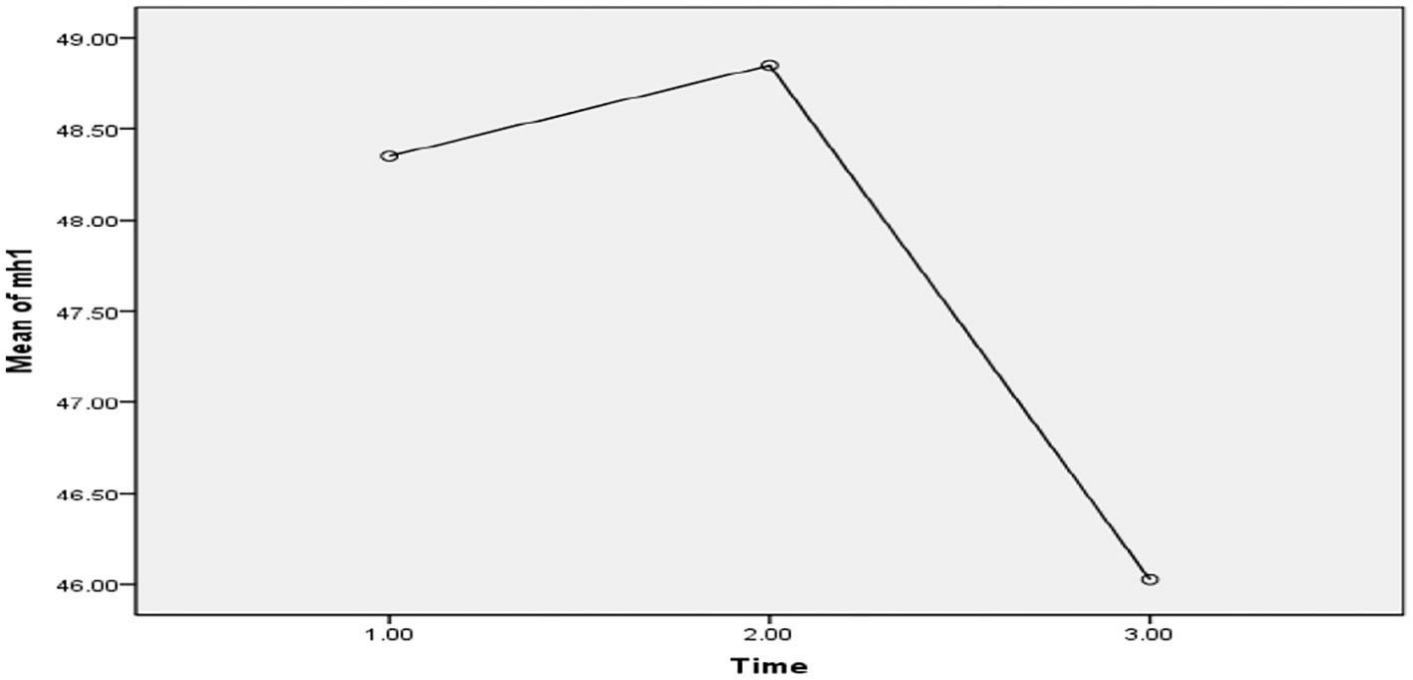
Illustrative mean difference of mental health between the pretesting phase (T-0), posttesting phase (T-1), and follow-up phase (T-2) in Pakistan students during the COVID-19 epidemic lockdown (*N* = 40).

**Figure 8:**
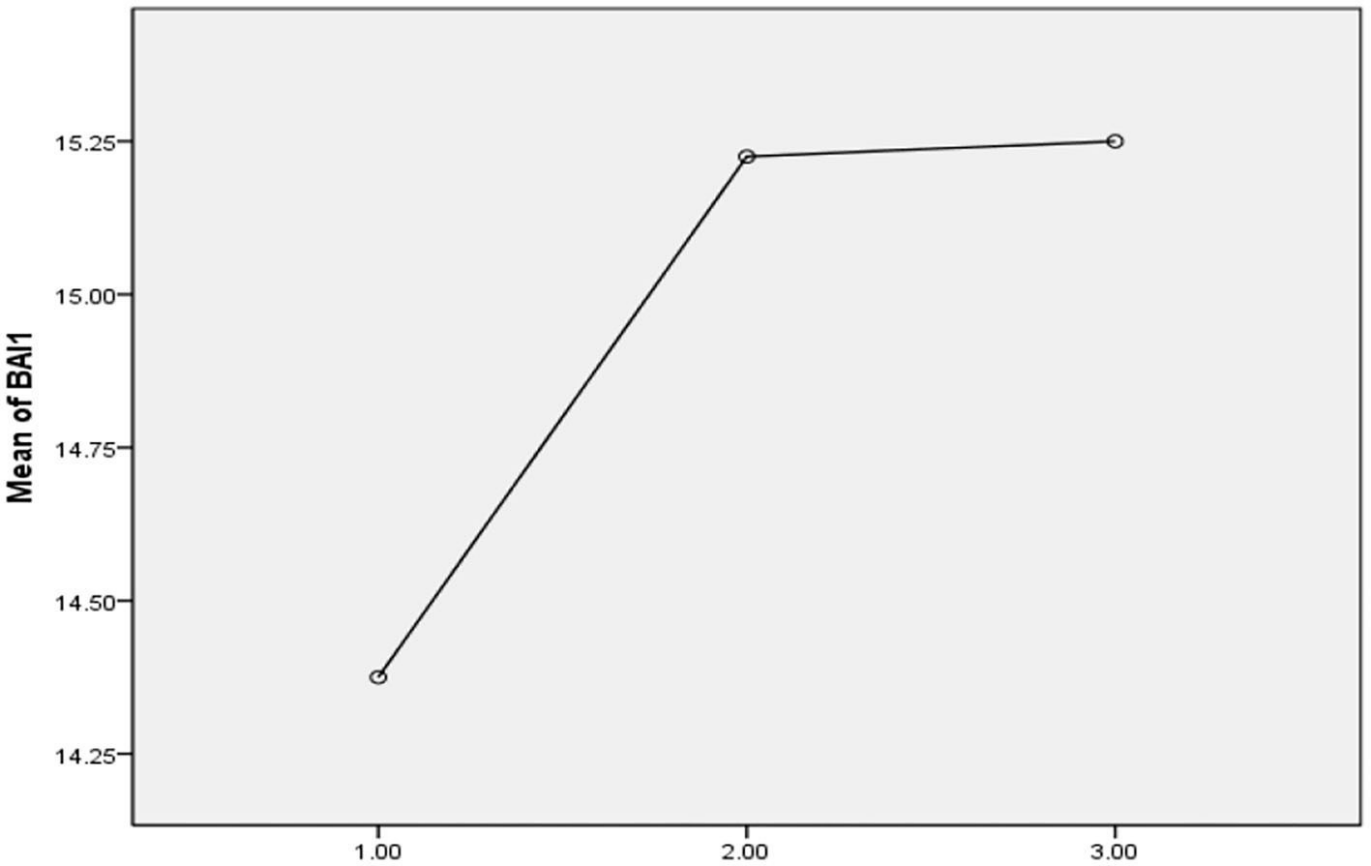
Illustrative mean difference of anxiety disorder between the pretesting phase (T-0), posttesting phase (T-1), and follow-up phase (T-2) in Pakistan students during the COVID-19 epidemic lockdown (*N* = 40).

**Figure 9:**
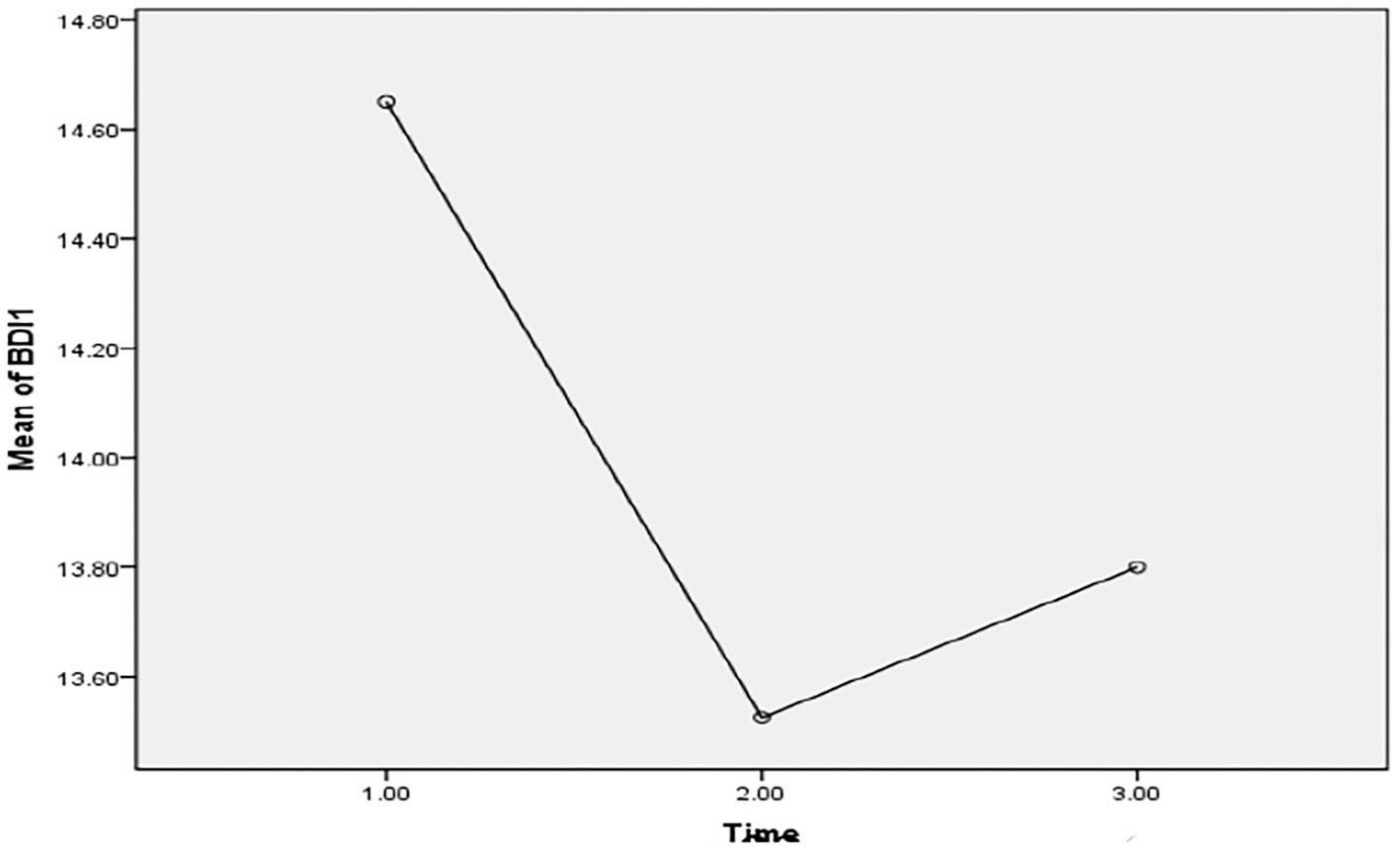
Illustrative mean difference of depression disorder between the pretesting phase (T-0), posttesting phase (T-1), and follow-up phase (T-2) in Pakistan students during the COVID-19 Epidemic lockdown (*N* = 40).

**Figure 10:**
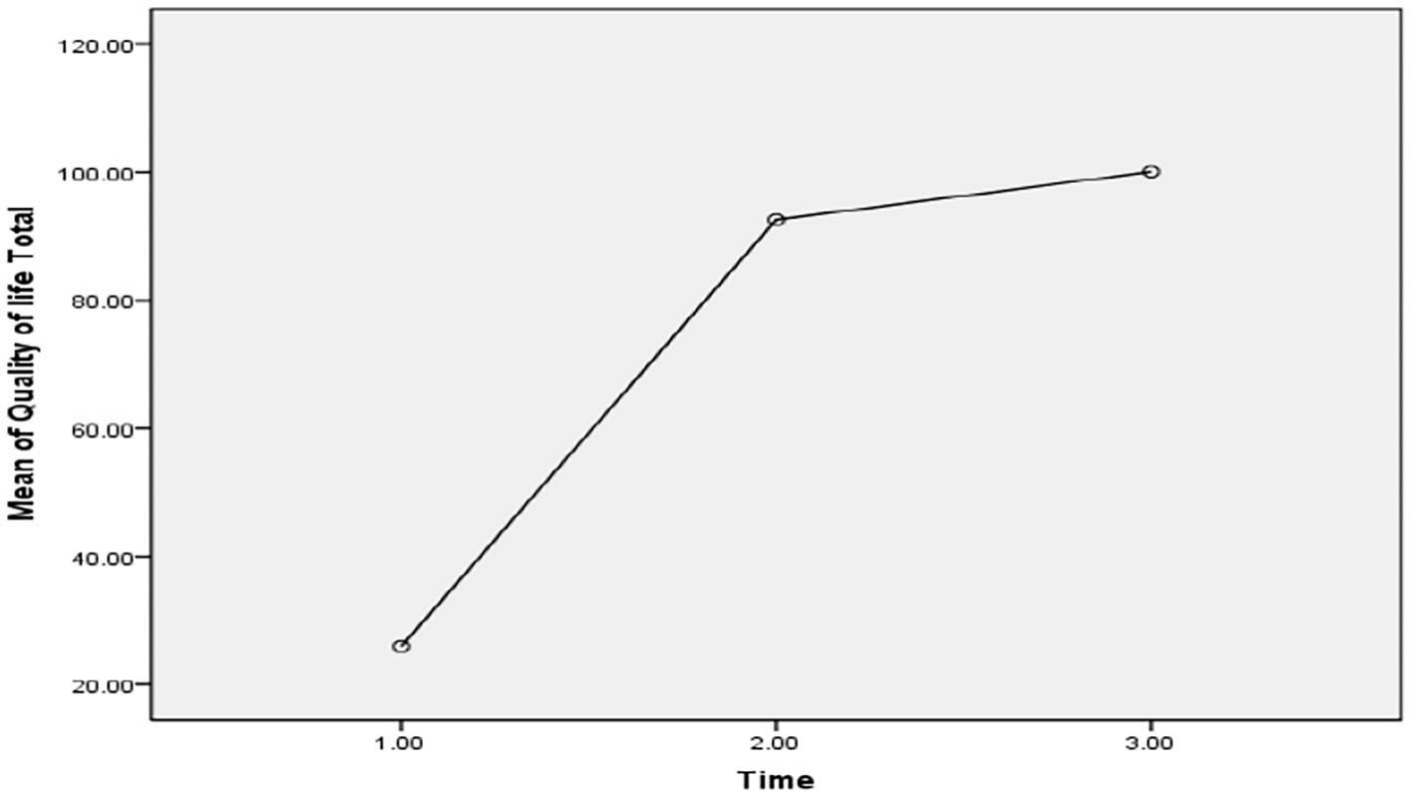
Illustrative mean difference of quality of life between the pretesting phase (T-0), posttesting phase (T-1), and follow-up phase (T-2) in Pakistan students during the COVID-19 epidemic lockdown (*N* = 40).

In Table 1, the results of the present study also demonstrated that university students were reported to have different levels of anxiety and depression during the full and partial lockdown in Pakistan. It revealed that partial lockdown is more effective in improving the quality of life and reducing symptoms of anxiety in comparison with full lockdown in the sample of Pakistani students. The results of the present study did not support the first hypothesis of the present study. The results showed that there was a decrease in the number of students who reported as having a normal level of anxiety in the initial phase (T-0), whereas the level of depression remained more or less constant throughout the study. Concurrently, Hypothesis 2 of the study was also rejected as the findings in Table 2 demonstrated that a full lockdown could be considered more appropriate to improve the quality of life, depression disorder, and mental health compared with partial lockdown during the COVID-19 epidemic (as evidenced by Figures 1, 2, 7–10 and Table 2). Furthermore, findings of the study revealed that a full lockdown significantly increased anxiety disorders in university students, whereas in partial lockdown, although there was a slight significant improvement in the quality of life, nevertheless, mental health also decreased in addition to an increase in anxiety and depression disorders (see Figures 1, 2, 7–10 and Table 2). The outcomes helped in achieving the objective of the study, which was to assess and compare the effects of full and partial lockdown on mental health, quality of life, anxiety, and depression in Pakistan student population. Although partial (smart) lockdown showed lesser effectiveness than the full lockdown, this could be due the time period, and no existing research available on the comparison between the two could be a potential indication for comparison between different countries. As an increase in anxiety was consistent throughout the full and partial lockdown, it is also consistent with findings of some previous research (10, 54).

**Table 1:** For baseline data chi square analysis between level of anxiety and depression pretesting phase (T-0), posttesting phase (T-1), and follow-up phase (T-2) in Pakistan students during COVID-19 epidemic lockdown (*N* = 40).

	**Pretest**				**Posttest**				**Follow-up**			
	**Observed**	**Expected**	**χ2**	***p*-Value**	**Observed**	**Expected**	**χ2**	***p*-Value**	**Observed**	**Expected**	**χ2**	***p*-Value**
**Anxiety**												
Normal	18	10.0	9.40	0.024	16	10.0	5.60	0.13	15	10.0	4.20	0.24
Mild	8	10.0			8	10.0			9	10.0		
Moderate	5	10.0			6	10.0			6	10.0		
Severe	9	10.0			10	10.0			10	10.0		
**Depression**												
Normal	25	10.0	30.60	0.000	24	10.0	26.20	0.000	24	10.0	28.20	0.000
Mild	4	10.0			6	10.0			3	10.0		
Moderate	4	10.0			5	10.0			9	10.0		
Severe	7	10.0			5	10.0			4	10.0		

**Table 2:** Mean difference of anxiety disorder, depression, quality of life, and mental health between pretesting phase (T-0), posttesting phase (T-1), and follow-up phase (T-2) in Pakistan students during COVID-19 epidemic lockdown (*N* = 40).

**Variables**	**Pretest phase**		**Posttest phase**		**Follow-up test phase**			***p***	**η^2^**
	**(*****n** **=*** **40)**		**(*****n** **=*** **40)**		**(*****n** **=*** **40)**				
	**M**	**SD**	**M**	**SD**	**M**	**SD**	**F**		
Mental health	48.35	10.31	48.85	10.36	46.02	10.83	0.82	0.44	–
Anxiety	14.37	13.47	15.22	13.38	15.25	13.64	0.05	0.94	–
Depression	14.65	12.87	13.52	10.81	13.80	11.33	0.10	0.90	–
Quality of life	25.95	0.22	92.60	16.91	100.07	13.49	426.98	0.000	0.99

Similarly, results in Table 3 of the study indicated that those participants who had reported preexisting moderate and severe levels of depression and anxiety disorders during both lockdowns showed that their mental health deteriorated in comparison with those who had reported normal and mild level of depression and anxiety disorders in both lockdown phases (See Figures 1, 2, 7–9 and Table 2). Moreover, the findings of the study also indicated that all participants who reported preexisting anxiety and depression disorders during both lockdown phases had an increased quality of life. Thus, the findings facilitated in achieving the second aim of the study, which was to assess the effect of the different phases of the lockdown on students with preexisting anxiety and depression (see Figures 1, 2, 7–9 and Table 2). Both lockdown situations have their own positive or negative outcomes in humans globally. Similar in Pakistan context, full lock down appeared more beneficial and favorable to improve mental health, quality of life, and depression disorder in university students during the COVID-19 epidemic, but the prevalence of anxiety disorder was increased in university students. However, partial lockdown also improved mental health, but it reduced the quality of life as well as increased mental issues such as depression and anxiety in university students. These findings are also in line with that of studies of similar nature conducted on student samples (58, 66, 80).

**Table 3:** Mean difference of quality of life and mental health between pre-testing phase (T-0), post testing phase (T-1), and follow-up phase (T-2) with different level of anxiety and depression in Pakistan students during the COVID-19 epidemic lockdown (*N* = 40).

	**Pretesting phase (T-0)**			**Posttesting phase (T-1)**			**Follow-up testing phase (T-2)**			**BAI**		**Time**		**BAI** *time		
**Level of anxiety**	**M**	**SD**	***N***	**M**	**SD**	***N***	**M**	**SD**	***N***	***F***	***p*-value**	***F***	***p*-value**	***F***	***p*-value**	** $\eta_{\text{p}}^{2}$ **
**Quality of life**																
Normal	25.94	0.23	18	101.93	13.01	16	106.60	10.79	15	2.99	0.12	215.35	0.00	1.94	0.08	1.00
Mild	26.00	0.02	8	85.50	24.14	8	101.33	15.82	9							
Moderate	25.80	0.44	5	88.16	9.70	6	96.16	8.70	6							
Severe	26.00	0.01	9	86.00	13.78	10	91.50	13.35	10							
**Mental health**																
Normal	52.11	11.22	18	55.43	8.86	16	51.00	10.74	15	19.81	0.002	1.02	0.39	0.40	0.87	0.99
Mild	48.00	5.83	8	43.87	13.62	8	46.44	13.63	9							
Moderate	43.80	8.01	5	46.00	7.23	6	43.83	5.56	6							
Severe	43.66	10.94	9	44.00	5.49	10	39.50	7.41	10							
	**Pretesting phase (T-0)**			**Posttesting phase (T-1)**			**Follow-up testing phase (T-2)**			**BDI**		**Time**		**BDI** *time		
**Level of depression**	**M**	**SD**	***N***	**M**	**SD**	***N***	**M**	**SD**	***N***	***F***	***p*** **-value**	***F***	***p*** **-value**	***F***	***p*** **-value**	$\eta_{\text{p}}^{2}$
**Quality of life**																
Normal	25.96	0.20	25	95.54	19.28	24	105.58	11.38	24	2.82	0.12	210.46	0.00	1.21	0.30	0.98
Mild	26.00	0.00	4	89.16	10.98	6	100.00	7.00	3							
Moderate	26.00	0.00	4	83.60	5.94	5	90.55	13.29	9							
Severe	25.85	0.37	7	91.60	17.05	5	88.50	13.22	4							
**Mental health**																
Normal	51.36	10.44	25	50.95	11.81	24	51.00	9.13	24	24.31	0.00	1.90	0.18	0.49	0.80	0.99
Mild	51.00	6.37	4	51.83	4.30	6	50.66	2.30	3							
Moderate	43.00	7.25	4	41.00	4.52	5	37.11	6.19	9							
Severe	39.14	6.96	7	43.00	6.36	5	32.75	9.42	4							

*BAI, Back Anxiety Inventory; BDI, Back Depression Inventory; QOL, Quality of life; MH, Mental Health*.

The current results clarified the present study's aforementioned objectives. They are also consistent with the findings of other studies with similar subject matter (11, 54, 58, 66, 80). Unfortunately, the few studies conducted on the effectiveness of both partial and full lockdown on mental health issues were not consistent because of either different samples or were more of a review, in general, rather than an empirical study (81, 82). However, recently many governments of different countries have been taking steps in implementing partial lockdown to handle the COVID-19 pandemic crisis such as many European countries like Germany, Rome, and Calabria (83). The results based on statistics suggest that partial lockdown may be better in controlling the spread of the virus while sustaining economic conditions (48, 59, 84–88). The findings of the present study highlight the effects of a stretched-out lockdown on a student's mental health (50, 89–93). Nevertheless, this should be further studied in a larger setting to check the effect of both full and partial lockdown on different populations in future studies on a larger sample.

Findings of the current study can help out in comprehending the eminent need of interventional strategies to cater to the mental health issues students are facing as a result of lockdown in Pakistan. No doubt, the economic outcomes are merely too large to plan a full lockdown in Pakistan, especially when majority of the people live below the poverty line. However, controlling and mitigating only the spread of the virus while ignoring the severe mental health consequences as a result are not permanent solutions, and authorities should devise strategies such as online counseling sessions or a reduced number of physical classes with odd–even number of students for them to relieve their stress and anxiety.

## Future Implications

The present study is currently one of its kind as it has tried not only to assess the effect of lockdown on psychological health of students but also has tried to incorporate the effect of both full and partial lockdown on a student's psychological health. The findings call for immediate action by policymakers to devise mental health interventions for student's mental health. Additionally, the study can also prove beneficial for authorities to design a lockdown system while taking into consideration the effects of lockdown on the mental health of student population in Pakistan.

## Limitations and Suggestions

1. The study though was effective in comparing the effect of full and partial lockdown on a student's psychological health, and the sample size was relatively small since the access was mostly online and limited. Future studies should incorporate a larger sample to encapsulate the findings.
2. Although data were collected as soon as the lockdown was in effect, however, since the lockdown already started, the students' psychological health was already affected. It would have been better if initial data could have been collected from the time universities were still open.
3. Another limitation was the difference in timing of full and partial lockdown, which could have an effect on the overall findings since the full lock down in Pakistan was for a shorter period, while partial lockdown was for months. It would be interesting for future studies to compare data between the countries with full lockdown for the same period with that of partial lockdown in Pakistan.

## Conclusion

The results of the present study illustrate that university students are considered a vulnerable populace, and particular interventions and preventions are required to protect and improve their mental health and quality of life during the epidemic globally. It would also be very interesting to examine the psychological influence of the following waves of epidemic because of the persistence of the epidemic's stressful experience in Pakistan. Additionally, the findings of the present study are crucial in assessing the effect of lockdown on student's psychological mental health and quality of life. The study can be used to plan future lockdown accordingly and implementation of mental health interventions to improve mental health and quality of life of affected students and those with preexisting mental health problems. This study concluded that the COVID-19 epidemic lockdown was a more effective and preventive tool against COVID-19 to improve quality of life, mental health, and depression compared with partial lockdown in Pakistan students. Findings of this study suggested to keep full lockdown for a shorter period of time in a vulnerable university to tackle the COVID-19 crisis. This current article proposes a preventive model that helps reduce students' mental health and quality of life challenges amid partial and complete lockdowns of the COVID-19 pandemic. Illness perception develops into individuals' mental disorders, such as psychological disorders, depression, and anxiety problems, that can reduce an individual's mental health. Ultimately, it influences an individuals' quality of life. As a result, there is a need for crucial preventive measures for the ongoing pandemic to conduct clinical investigations to address depression, anxiety, and mental disorders. This study's findings offer helpful insights and recommend practical steps to evaluate the individuals' mental health issues caused by the present pandemic. The managerial and clinical preventive strategies suggest clinical examinations to combat this lethal pandemic worldwide. The study results recommend that health professionals formulate a preventative strategy to educate people to follow preventive measures. The findings suggest promoting safety education and healthcare facilities amid the COVID-19's wide-ranging crisis. The study outcomes climax the vital preventive strategies to deal with the mental health challenges in this current pandemic.

## Data Availability Statement

The original contributions presented in the study are included in the article/supplementary material, further inquiries can be directed to the corresponding author/s.

## Ethics Statement

The studies involving human participants were reviewed and approved by Foundation University of Islamabad. The patients/participants provided their written informed consent to participate in this study.

## Author Contributions

All authors listed have made a substantial, direct, and intellectual contribution to the work and approved it for publication.

## Conflict of Interest

The authors declare that the research was conducted in the absence of any commercial or financial relationships that could be construed as a potential conflict of interest.

## Publisher's Note

All claims expressed in this article are solely those of the authors and do not necessarily represent those of their affiliated organizations, or those of the publisher, the editors and the reviewers. Any product that may be evaluated in this article, or claim that may be made by its manufacturer, is not guaranteed or endorsed by the publisher.
